# Editorial: Hormone Action and Signal Transduction in Endocrine Physiology and Disease

**DOI:** 10.3389/fendo.2020.00589

**Published:** 2020-08-27

**Authors:** Tamas Balla, László Hunyady

**Affiliations:** ^1^Eunice Kennedy Shriver, National Institute of Child Health and Human Development, National Institutes of Health, Bethesda, MD, United States; ^2^Department of Physiology, Faculty of Medicine, Semmelweis University, Budapest, Hungary

**Keywords:** signal transduction, ACTH, G protein, beta-arrestin, sertolli cells, gonadotroph cell, myometrium contractility, TSH receptor

The Frontiers Research Topic on “Hormone Action and Signal Transduction in Endocrine Physiology and Disease” is a collection of 10 papers inspired by the meeting held at Semmelweis University Budapest, August 16-17, 2018 in memory of Kevin J. Catt who passed away on October 1, 2017. Dr. Catt was a notable scientist who worked in areas related to receptors of protein and peptide hormones and their actions via various signal transduction mechanisms in many tissues. His interest was focused on the hypothalamo-adrenal axis as well as on the hormonal control of reproductive organs. The collection of chapters authored by Dr. Catt's disciples who spent time under his mentorship reflects the legacy of Dr. Catt in these research areas. The Authors are well-established and highly accomplished scientists who work on receptors, their signaling modalities and on biological effects of hormones and neurotransmitters. Although not a former trainee, a close collaborator of Dr. Catt has also contributed to this collection.

The chapters cover endocrine related functions in several organs including hypothalamus, pituitary and adrenal gland, reproductive organs, fetal signals, and ovarian tumors. They summarize recent advances and new discoveries on the functions of hormones, their signaling pathways and their impact on physiology as well as on pathology of various neuro- and somatic diseases.

Four of the Chapters focus on the hypothalamo-adrenal stress regulatory axis.

The Chapter by Antoni gives a historical perspective on the role of vasopressinergic neurons located in the supraoptic and paraventricular nuclei in the control of ACTH secretion from the anterior pituitary. This review covers an important topic, namely conditions under which the feed-back inhibition by glucocorticoid hormones in pituitary corticotrophs is bypassed (termed glucocorticoid escape) and the role of vasopressinergic neurons in the process.

The Chapter by Parra-Mercado et al. from the Hauger and Olivares-Reyes laboratories investigates the signaling properties of the corticotropin releasing factor receptor (CRF1R) in heterologous expression systems. This work teases out the contribution of tyrosine kinases, such as Src and EGF receptors as well as the known antiapoptotic phosphatidylinositol 3-kinase (PI3K) pathways in the control of ERK1/2 phosphorylation, the main signaling hub of CRF1R activation.

The Chapter by Clark and Chan. covers ACTH receptors (MC2R) that initiate activation and signaling in the adrenal cortex. They highlight the importance of the adrenal accessory protein, MRAP, which is essential for MC2R expression and its trafficking to the cell surface. The review discusses the control of mRNA and protein expression of MC2R and MRAP by ACTH, as well as the role of N-glycosylation in the stabilization of the MRAP homodimer. The dynamic association between MC2R and MRAP and its impact on ACTH receptor desensitization also receives ample coverage. The role of these processes in the control of adrenal zonation and cell renewal as they relate to adrenal diseases, cancer, and aging are discussed in detail.

The Chapter by Turu et al. from the group of László Hunyady focuses on the structure-function relationship of the AT1 angiotensin receptor, the main controller of mineralocorticoid secretion from the adrenal cortex. The authors discuss the structural features of the receptor that are critical for G protein coupling and activation as well as for β-arrestin binding during ligand engagement. They also highlight the fact that different ligands can induce conformational changes that selectively favor interaction of the receptor with their distinct protein binding partners mediating various aspects of their signaling. These findings offer new opportunities for pharmacological modulation of G protein and β-arrestin mediated signaling effects of G-protein coupled receptors independently.

Five chapters cover research areas related to the reproductive system. The central role of FSH, acting in concert with androgens in shaping pulsatile LH secretion is a major topic of interest. The effects of these hormones on Sertoli cell gene expression receive in-depth coverage and so does the Involvement of paracrine signals that are essential for the progress of spermatogenesis.

Bjelobaba et al. from the Stojilkovic group describe single cell RNA profiling studies from pituitary cells that revealed that gonadotroph cells display uniques expression patterns of osteopontin and its sibling, Dentin matrix protein 1. The authors discuss the implication of these factors in defining the tissue architecture of the anterior pituitary and its possible response to changes in the reproductive cycle and during tumorigenesis.

Meroni et al. from the Cigorraga group present an extensive overview on the hormones and locally produced factors as well as their signal transduction pathways and molecular mechanisms that control Sertoli cells proliferation and maturation. These data highlight mechanisms by which the number of Sertoli cells is controlled, which is an important determinant of the spermatogenic capacity of the testis. The review also discusses possible harmful effects caused by exposure to pollutants and frequently used pharmaceutical products in premature decrease in Sertoli cell number and impaired spermatogenesis.

Mendelson et al. review the factors that control myometrium contractility during pregnancy, with special emphasis on changes that contribute to the initiation of labor in humans and rodents. The authors discuss mechanisms by which progersterone, acting through various progesterone receptor isoforms, maintains myometrial quiescence during pregnancy. They also describe changes that underlie the decline in progesterone receptor function, leading to initiation of labor and the roles of miRNAs regulated by estradiol and progesterone in these processes.

Dufau and Kavarthapu describe the essential role of GRTH/DDX25, an RNA helicase, in the process of spermatogenesis. This helicase, discovered in the Dufau laboratory, is responsible for the elongation process of round spermatocytes. The biochemical events and various genes that are involved (mRNA transport/ translation) by the GRTH/DDX25 helicase receive ample coverage. The regulation of GRTH by androgens through an increases in the level of the transcription factor GCNF in round spermatids establishes a paracrine interplay between Sertoli cells and germ cells. Inhibition of the phosphorylation of GRTH is currently being pursued by this group as a potential orally active and reversible male contraceptive strategy.

Tocci et al. from the group of Bagnato review the importance of Endothelin-1 Receptor (ET-1R) and its signaling in the development of ovarian cancer and discuss new therapeutic approaches interfering with this system. They describe how ET-1R and β-arrestin-1 mediated signaling contribute to tumor progression in the ovary and describe combinatorial approaches as potential means to block these pathways.

Lastly, Latif et al. from the Davies laboratory describe recent progresses in developing small molecules that could stimulate TSH receptors, specifically engaging the Gq-phospholipase C pathway for pharmacological control of thyroid hormone secretion. They authors review the properties of a new agonist that activates cross-talk between the Ca^2+^ and cAMP messenger systems and discuss its potential use in controlling proliferation of thyroid cells.

The collection of these chapters not only honors the Dr. Catt and his scientific legacy but will undoubtedly inspire both new investigators and established scientists to advance these important research areas with their own research.

## Author Contributions

TB and LH contributed to the preparation of the manuscript. All authors contributed to the article and approved the submitted version.

## Conflict of Interest

The authors declare that the research was conducted in the absence of any commercial or financial relationships that could be construed as a potential conflict of interest.

